# Ethyl Pyruvate Attenuates Microglial NLRP3 Inflammasome Activation via Inhibition of HMGB1/NF-κB/miR-223 Signaling

**DOI:** 10.3390/antiox10050745

**Published:** 2021-05-08

**Authors:** Melis Olcum, Kemal Ugur Tufekci, Devrim Yagmur Durur, Bora Tastan, Irem Nur Gokbayrak, Kursad Genc, Sermin Genc

**Affiliations:** 1 Izmir Biomedicine and Genome Center, Izmir 35340, Turkey; melis.olcum@ibg.edu.tr (M.O.); devrimyagmur.durur@msfr.ibg.edu.tr (D.Y.D.); bora.tastan@msfr.ibg.edu.tr (B.T.); 2 Vocational School of Health Services, Izmir Democracy University, Izmir 35290, Turkey; kemalugur.tufekci@idu.edu.tr; 3 Izmir International Biomedicine and Genome Institute, Dokuz Eylul University, Izmir 35340, Turkey; 4 Department of Neuroscience, Health Science Institute, Dokuz Eylul University, Izmir 35340, Turkey; iremnur.gokbayrakatay@ogr.deu.edu.tr (I.N.G.); kemal.genc@deu.edu.tr (K.G.)

**Keywords:** ethyl pyruvate, microglia, NLRP3 inflammasome, microRNA, HMGB1, NF-κB

## Abstract

Ethyl pyruvate is a molecule with anti-inflammatory and pro-metabolic effects. Ethyl pyruvate has been shown to ameliorate the clinical and pathological findings of neurodegenerative diseases such as Alzheimer’s and Parkinson’s Diseases in rodents. Its anti-inflammatory and neuroprotective effects are widely investigated in animal and cellular models. Our study aimed to investigate the mechanism of the impact of Ethyl pyruvate on NLRP3 inflammasome activation in the N9 microglial cell line. Our results indicated that ethyl pyruvate significantly suppressed LPS and ATP-induced NLRP3 inflammasome activation, decreased active caspase-1 level, secretion of IL-1β and IL-18 cytokines, and reduced the level of pyroptotic cell death resulting from inflammasome activation. Furthermore, ethyl pyruvate reduced the formation of total and mitochondrial ROS and suppressed inflammasome-induced HMGB1 upregulation and nuclear NF-κB translocation and reversed the inflammasome activation-induced miRNA expression profile for miR-223 in N9 cells. Our study suggests that ethyl pyruvate effectively suppresses the NLRP3 inflammasome activation in microglial cells regulation by miR-223 and NF-κB/HMGB1 axis.

## 1. Introduction

Microglial cells are the primary innate immune component of the central nervous system (CNS), which make up 10–15% of the brain [1]. They are mainly involved in maintaining tissue homeostasis by constantly scavenging the environment for possible threats and initiating the proper inflammatory responses, including inflammasomes. Microglial cells are the primary source of inflammasomes in the central nervous system [2]. Microglial inflammasome activation can be triggered by lipopolysaccharide (LPS), alpha-synuclein, prion peptide, and ATP, which then causes the secretion of pro-inflammatory cytokines and reactive oxygen species [3].

Cytoplasmic pattern recognition receptors (PRRs) are the first to encounter and recognize foreign bodies and pathogens as they enter the cells [4]. NOD-like receptor (NLR) family of PRRs can recognize damage-associated molecular patterns (DAMPs) and pathogen-associated molecular patterns (PAMPs) to activate the appropriate inflammatory response [5]. NLRP3 inflammasome complex is one of the inflammatory responsive complexes of the NLR family, which is activated via ATP and leads to pro-inflammatory cytokine release and pyroptotic cell death [6]. The proteolytic multiprotein complex structure of the NLRP3 inflammasome complex induces the caspase-1 enzyme to get activated and causes the secretion of IL-1β and IL-18 outside the cell [7]. Furthermore, autocatalytic activation of caspase-1 leads to activation of Gasdermin D (GSDMD), a pore-forming protein initiating pyroptosis [8]. NLRP3 inflammasome is regulated transcriptionally and post-translationally. miR-223 is a hematopoietic microRNA(miRNA) that plays a role in myeloid differentiation and dendritic cell activation [9,10]. NLRP3 inflammasome regulation by miR-223 was first shown by Bauerfeind et al. in 2012 [11]. Moreover, the role of miR-223 has been shown in several neurodegenerative diseases, such as multiple sclerosis and Alzheimer’s disease.

Pyruvate, a potent protective molecule in many organ systems, is an intermediate molecule of glycolysis; however, its limited stability restricts wide use in clinics. As its derivative, ethyl pyruvate (EP) is a lipophilic and highly stable pyruvate ester [12], with neuroprotective, anti-inflammatory, and anti-apoptotic properties [13,14]. In the same manner as pyruvate, in vivo and in vitro use of EP reportedly reduces the tissue damage in sepsis, hemorrhagic shock, stroke, inflammation [15,16]. Its anti-inflammatory action involves suppression of ROS production [17] and NO release in activated primary microglia in vitro [18]; and suppression of IL-6 [19] and TNF-α in LPS-induced BV2 cells [20]. Therefore, in this study, we aimed to investigate EP’s effect on NLRP3 inflammasome activation in microglial cells. Here, we demonstrated that EP suppressed LPS and ATP-induced NLRP3 inflammasome activation via miR-223 regulation on the NF-κB/HMGB1 axis.

## 2. Materials and Methods

### 2.1. Cell Culture and Treatments

Our study used the N9 microglial cell line, which is extensively used in microglia research as it is a successful substitute for primary microglia culture [21]. N9 cell line is very similar to primary cells in terms of the inflammatory mediator activation upon LPS treatment, morphology, migratory ability, and phagocytosis ability [22]. N9 mouse microglial cell line used in this study was provided by Paola Ricciardi-Castagnoli (Cellular Pharmacology Center, Milan, Italy) and cultured in RPMI 1640 medium (Gibco, Thermo Fisher Scientific, Waltham, MA, USA) supplemented with 10% fetal bovine serum (Gibco, Thermo Fisher Scientific, Waltham, MA, USA), 100 U/mL penicillin, and 100 μg/mL streptomycin (Gibco, Thermo Fisher Scientific, Waltham, MA, USA). For the inflammasome activation, cells were treated with ATP (Sigma Aldrich, St Louis, MO, USA ) (5 mM, 1 h) after incubation with ultra-pure LPS (Invivogen, San Diego, CA, USA) (1 µg/mL, 4 h). The cells were incubated with EP at a concentration of 1–100 mM to observe EP’s effect on inflammasome activation in microglial cells stimulated with LPS and ATP. After determining the effects of EP on cell viability in N9 cells, 10 mM concentration was selected for EP treatments for further experiments.

### 2.2. Cell Viability Assessment with Presto Blue

Cell viability was measured with Presto Blue cell viability reagent (Invitrogen, Thermo Fisher Scientific, Waltham, MA, USA). For this, 5 × 10^3^ N9 cells were seeded in a 96-well plate 24 h before treatments. Ten microliters of Presto Blue cell viability reagent (Thermo Fisher Scientific, Waltham, MA, USA) were added to the cells at the end of the treatment. The cells were then incubated at 37 °C for 30 min. Finally, the fluorescence was measured at an excitation of 560 nm and emission of 590 nm using a microplate reader (Varioskan Flash, Thermo, Waltham, MA, USA). Relative viability was calculated compared to the untreated control.

### 2.3. Propidium Iodide Staining

The effect of EP on LPS- and ATP-induced pyroptotic cell death due to inflammasome activation in microglial cells was determined by propidium iodide (PI) staining. At the end of the incubation, 50 μg/mL PI dye (Thermo Fisher Scientific, Waltham, MA, USA) was added to each well and imaged through a fluorescent microscope, and the wells were photographed. PI-positive cells were counted from these photographs and analyzed [23].

### 2.4. ASC Speck Staining

Microglial cells were seeded into 25 cm^2^ flasks with a density of 1 × 10^6^ cells per flask. Following the treatment, microglial cells were fixed with 4% paraformaldehyde at 37 °C for 15 min and washed with PBS twice. Permeabilization and blocking were performed with PBS containing 10% FBS and 0.5% Triton-X-100 at 3 °C for 30 min. Cells were incubated with ASC primary antibody (Santa Cruz Biotechnology, sc-33958, 1:100 diluted) overnight, followed by Alexa fluor-594 conjugated anti-goat antibody (Jackson Immunoresearch, 705-585-003, 1:1000) for one h. ASC speck images were acquired with LSM 880 Confocal microscopy (Zeiss, Germany). ASC speck positive cells were counted by ImageJ (National Institutes of Health, Bethesda, MD, USA) [24], and data were given as the percentage of total cells [23].

### 2.5. ELISA

The effects of EP on inflammasome activation in microglial cells were assessed by determining IL-1β and IL-18 cytokine levels. Commercial sandwich ELISA (Ebioscience, Vienna, Austria) kits were used according to the manufacturer’s protocol to analyze IL-1β and IL-18 cytokines. The photometric measurements were performed at 450 nm using a microplate reader, Varioskan Flash (Thermo Scientific, Waltham, MA, USA).

### 2.6. Detection of Intracellular ROS

Intracellular ROS was measured using CM-H_2_DCFDA (Invitrogen, Carlsbad, CA, USA) fluorescent dye. Following the treatments, microglial cells were treated with CM-H_2_DCFDA for 15 min at 37 °C. The fluorometric measurements were performed according to the manufacturer’s protocol using a microplate reader, Varioskan Flash (Thermo Scientific, Waltham, MA, USA) [23].

### 2.7. Detection of Mitochondrial ROS

Mitochondrial ROS was detected with MitoSOX (Invitrogen, Carlsbad, CA, USA), a red fluorogenic dye specifically targeting mitochondria in living cells. For the fluorometric assay, treated microglial cells were incubated with MitoSOX (5 μM) for 15 min at 37 °C. Absorbance values were measured according to the manufacturer’s instructions using a microplate reader Varioskan (Thermo Scientific, Waltham, MA, USA). For immunofluorescence assay, treated cells were incubated with 5 μM MitoSOX for 15 min and 0.1 μM Hoechst at 37 °C for nucleus staining. Images were obtained under an inverted fluorescent microscope (Olympus IX-71, Olympus, Japan) [23].

### 2.8. Analysis of Mitochondrial Membrane Potential

According to the manufacturer’s protocols, the mitochondrial membrane potential of microglial cells was analyzed using JC-1 stain (Thermo Scientific, Waltham, MA USA). JC-1, a cationic stain accumulating on the mitochondrial membrane, yields red fluorescence in healthy mitochondria, whereas it displays green fluorescence in depolarized or damaged mitochondria. Treated microglial cells were incubated with 2.5 μg/mL JC-1 at 37 °C in the dark. A FACS Canto II analyzer using a 488 nm laser (Becton Dickinson, Franklin Lakes, NJ, USA) was used for assessing the mitochondrial membrane potential. The red/green fluorescence intensity ratio was used to express the change in mitochondrial membrane potential [23].

### 2.9. Western Blotting

The cell lysate and supernatants’ protein content were extracted using RIPA buffer (50 mM Tris-HCl, pH 7.4, 150 mM NaCl, 0.25% deoxycholic acid, 1% Nonidet P-40, 1 mM EDTA), including the protease and phosphatase inhibitor (Thermo Scientific, Waltham, MA, USA). For nuclear and cytosolic fractions, NE-PER Nuclear and Cytoplasmic Extraction Reagents (Thermo Scientific, Waltham, MA USA) were used according to the manufacturer’s instructions. The extracted proteins were stored at −80 °C. Protein samples were separated by 10% sodium dodecyl sulfate-polyacrylamide gel electrophoresis (SDS-PAGE). Then, the gels were transferred to the polyvinylidene difluoride (PVDF) membrane and incubated in a blocking buffer containing 5% milk after transfer. The membranes were then incubated with primary antibodies (Table 1). After the washing steps, the samples were incubated with the secondary antibody labeled with horseradish peroxidase (HRP), and after washing, the membrane was visualized by the chemiluminescence method. The obtained band densities were evaluated [23].

### 2.10. Quantitative PCR

RNA from the cells was isolated with the RNAeasy mini kit (Qiagen, Hilden, Germany), and cDNA synthesis was performed. PCR was performed using qPCR master mix containing SYBR green with primers of GAPDH and pro-inflammatory molecules (IL-1β, IL-18, NLRP3) (Applied Biosystems 7500 Fast, Foster City, CA, USA) (Table 2). For the detection of miRNAs, miR-223 and endogenous control U6 primer assays were utilized (Qiagen, Hilden, Germany). The expression difference was analyzed by the ΔΔCt method with endogenous normalization to GAPDH or U6 [23].

### 2.11. Transfection of miRNA Inhibitors

MiR-223 antagomiR and negative controls were purchased from Qiagen. N9 cells were transfected 24 h after seeding in cell culture plates with a density of 1.5 × 10^4^ cells per well in a 6-well plate. According to the manufacturer’s protocol, transfection of cells with miR-223 antagomiR and negative control oligomers was performed using the HiPerFect transfection reagent (Qiagen, Hilden, Germany). The final concentration of the antagomiR and negative controls was 50 nM. After 24 h, media from each well was replaced with inflammasome activation media and EP administration. Following transfection and treatments, qPCR was performed to assess inflammasome markers.

### 2.12. Statistical Analysis

The statistical analysis of the results was done using Graphpad Prism 8.4 software. Data are presented as Mean ± Standard Error of the Mean. Mann−Whitney U test was used to compare between groups. The significance level was accepted as *p* < 0.05 in all evaluations.

## 3. Results

### 3.1. EP Decreased IL-1β and IL-18 on Both mRNA and Protein Levels

**Figure 1:**
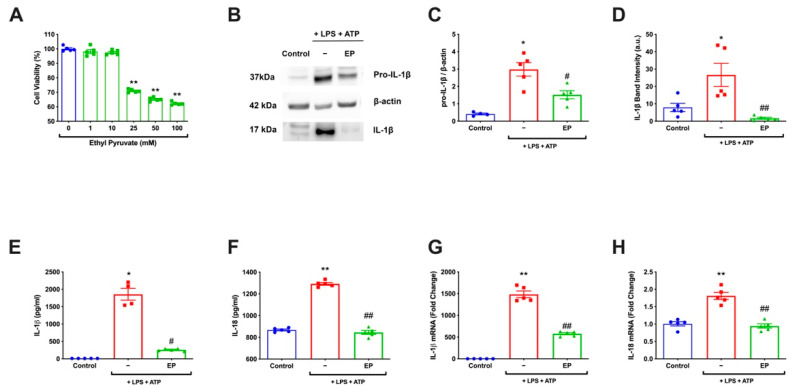
EP reduced mRNA and protein levels of IL-1β and IL-18. N9 microglial cells were pretreated with EP (10 mM) for one hour, then treated with LPS (1 μg/mL) for four hours and ATP (5 mM) for 1 h. (**A**) The toxicity of EP was determined. (**B**–**D**) Protein levels of pro-IL-1β and secreted IL-1β were reduced by EP compared to LPS- and ATP-induced cells. (**E**,**F**) The suppressor effect of EP on secreted IL-1β and IL-18 was measured with ELISA. (**G**,**H**) mRNA levels of IL-1β and IL-18 reduced by EP pre-treatment compared to LPS and ATP induction group. Data are presented as mean ± S.E.M, n = 5. * *p* < 0.05, ** *p* < 0.01 compared to control and # *p* < 0.05, ## *p* < 0.01 compared to LPS- and ATP-induced cells.

Firstly, EP’s effect on basal cell death at a concentration of 1–100 mM was investigated. According to our analysis, EP did not show cytotoxic effects on the cells in the range of 1–10 mM (Figure 1A) (** *p* < 0.01). Therefore, a 10 mM dose was selected for further experiments. To determine EP’s effect on inflammation markers, protein levels of IL-1β and IL-18 levels were detected. For IL-1β, both pro and mature forms were analyzed using western blot (Figure 1B). Pro-IL-1β densities showed a significant increase in the LPS and ATP group than the control (*p* = 0.0159). There was a statistically significant decrease in the EP pre-incubated group than the LPS and ATP group (*p* = 0.0159) (Figure 1C). When IL-1β amounts were compared, there was a statistically significant increase in the LPS and ATP group compared to the control group (*p* = 0.0317). With the 10 mM pre-incubation of EP, the rate of IL-1β increased with LPS and ATP decreased statistically (*p* = 0.0079) (Figure 1D). The protein levels of cytokines were also assessed by ELISA. Our results revealed that IL-1β and IL-18 levels in the control group were increased significantly in the LPS and ATP group compared to the control group (*p* = 0.016 and *p* = 0.008). In the EP pre-incubation group, it was found that IL-1β and IL-18 levels decreased statistically significantly compared to the LPS- and ATP-treated group. (*p* = 0.016 and *p* = 0.008) (Figure 1E, F). The effect of EP on the mRNA levels of IL-1β and IL-18, which are the markers of inflammasome activation, was investigated. A statistically significant increase was found in the LPS and ATP group compared to the control (*p* = 0.008). EP significantly suppressed LPS- and ATP-induced IL-1β and -18 mRNA levels (*p* = 0.008) (Figure 1G,H).

### 3.2. EP Diminished NLRP3 Protein Complex Formation by Reducing NLRP3, Caspase-1, and ASC Specks

**Figure 2:**
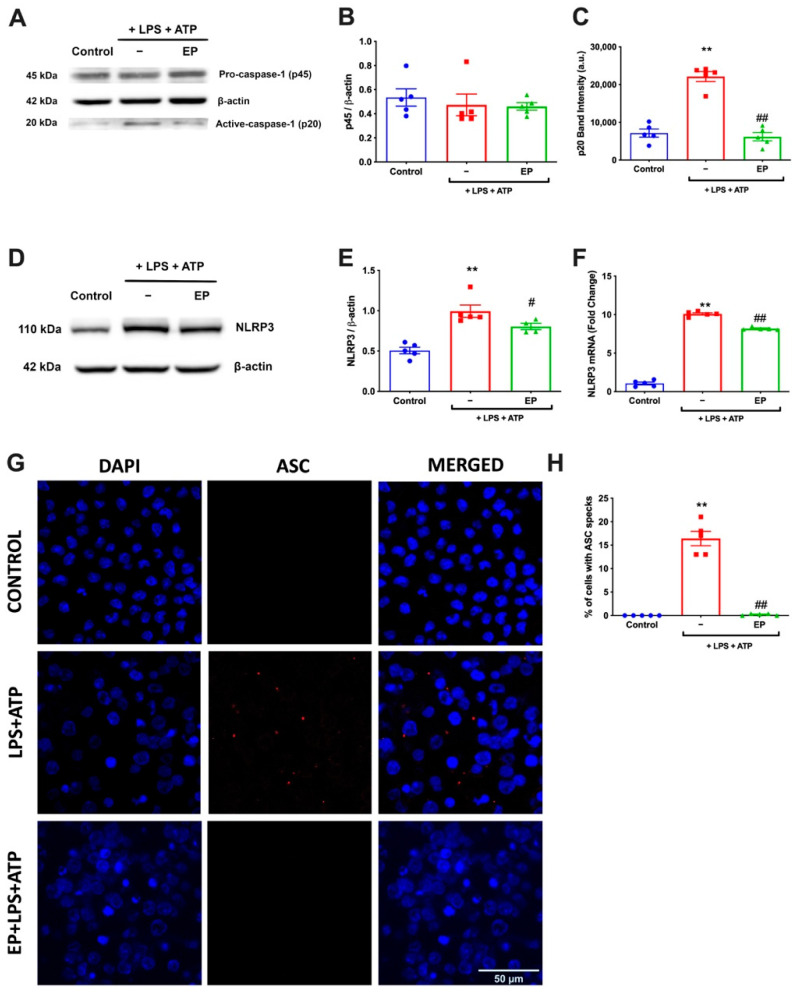
EP reduced NLRP3, caspase-1, and ASC speck formation. N9 microglial cells were pretreated with EP (10 mM) for one hour, then treated with LPS (1 μg/mL) for four hours, and ATP (5 mM) for 1 h. (**A**–**C**) Pro-caspase-1 shows no difference among groups. EP reduced cleaved caspase-1 in EP-pretreated cells compared to LPS- and ATP-induced cells. (**D**–**F**) EP suppressed NLRP3 on both protein and mRNA levels compared with LPS- and ATP-induced cells. (**G**) ASC speck formation was determined by confocal microscopy. (**H**) EP significantly prevented ASC speck formation compared to LPS- and ATP-induced cells. Data are presented as mean ± S.E.M, n = 5. ** *p* < 0.01 compared to control and # *p* < 0.05, ## *p* < 0.01 compared to LPS- and ATP-induced cells.

To determine the effects of EP on inflammasome activation, we have analyzed caspase-1 and NLRP3 levels. For caspase-1, we have measured the amounts of both pro and mature forms by western blot (Figure 2A). EP reduced the amount of active caspase-1 induced by LPS and ATP. On the other hand, there was no significant change in the amount of pro-caspase-1. Furthermore, NRLP3 expression was analyzed by both protein and mRNA levels. When NLRP3 protein amounts were compared, the EP pre-treatment group showed reduced levels of NLRP3 protein expression compared to the LPS and ATP group (Figure 2D,E). Moreover, the NLRP3 mRNA level was also reduced with the effect of EP pre-incubation compared to LPS and ATP induction (Figure 2F). EP significantly reduced the amount of NLRP3 increased with LPS and ATP. Regarding the formation of ASC specks as a crucial indicator of NLRP3 inflammasome activation, the percentage of cells with ASC specks (Figure 2G) was increased in LPS and ATP group compared to controls. With EP incubation, this was reduced (Figure 2H). The LPS and ATP treatment significantly increased ASC speck formation compared to the control group (*p* = 0.0004), while EP pre-treatment significantly reduced ASC speck formation compared to the LPS and ATP group (*p* = 0.0004).

### 3.3. EP Protected N9 Microglial Cells Against Pyroptotic Cell Death

**Figure 3:**
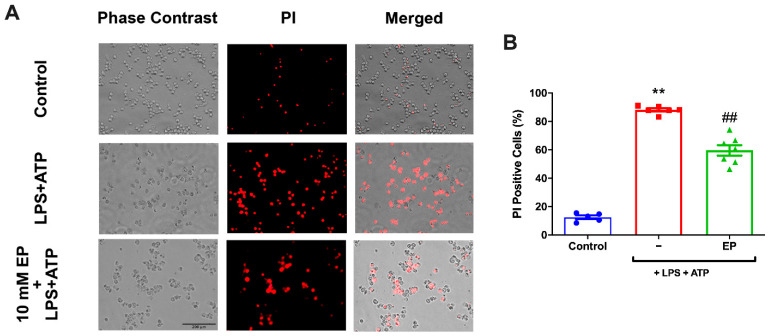
EP reduced pyroptotic cell death. N9 microglial cells were pretreated with EP (10 mM) for one hour, then treated with LPS (1 μg/mL) for four hours, and ATP (5 mM) for 1 h. (**A**,**B**) EP reduced pyroptotic cell death and decreased PI-positive cells. All the data are presented as mean ± S.E.M, n = 5. ** *p* < 0.01 compared to control and ## *p* < 0.01 compared to LPS- and ATP-induced cells.

The effect of EP on pyroptotic cell death in inflammasome activation was examined with PI staining (Figure 3A). The cytotoxicity rates determined by PI were significantly increased in LPS and ATP group compared to controls. With 10 mM EP pre-treatment, the PI-positive cell ratio was reduced (Figure 3B). The LPS and ATP group increased the cell death rate statistically significantly compared to the control group (*p* = 0.0079). Pre-incubation with different EP doses significantly decreased cellular death than the LPS and ATP group (*p* = 0.0025).

### 3.4. EP Ameliorated ROS Production and Restored Mitochondrial Membrane Potential

**Figure 4:**
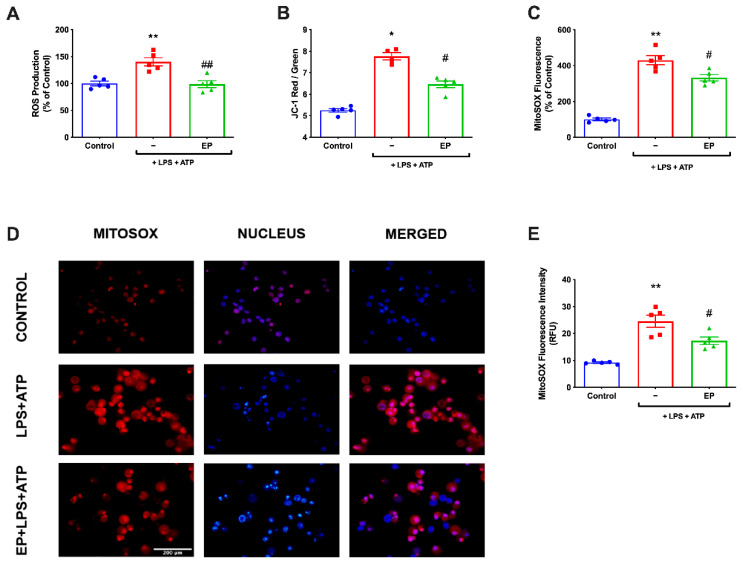
EP inhibited intracellular and mitochondrial ROS production and restored mitochondrial membrane potential. N9 microglial cells were pretreated with EP (10 mM) for one hour, then treated with LPS (1 μg/mL) for four hours, and ATP (5 mM) for 1 h. (**A**) EP pre-treatment reduced intracellular ROS production. (**B,C**) EP also decreased mitochondrial ROS production in EP-pretreated cells compared with LPS- and ATP-induced cells. (**D,E**) EP pre-treatment restored mitochondrial membrane potential. Data are presented as mean ± S.E.M, n = 5. * *p* < 0.05, ** *p* < 0.01 compared to control; # *p* < 0.05 compared to LPS- and ATP-induced cells and ## *p* < 0.01 compared to LPS- and ATP-induced cells.

Intercellular ROS was measured using CM-H2DCFDA in N9 microglial cells. While intracellular ROS level significantly increased in LPS- and ATP-induced cells, pre-treatment with EP reduced intracellular ROS production (Figure 4A). Furthermore, we examined mitochondrial ROS production and mitochondrial membrane potential in N9 microglial cells. Mitochondrial ROS level was increased with LPS and ATP treatment. However, EP pre-treatment significantly decreased mitochondrial ROS production to basal level (Figure 4B,C). The mitochondrial membrane potential was monitored with JC-1 staining. EP pre-treatment significantly restored membrane potential, disrupted with LPS and ATP treatment (Figure 4D,E).

### 3.5. EP Reduced NF-κB Activation and HMGB1 Expression Level

**Figure 5:**
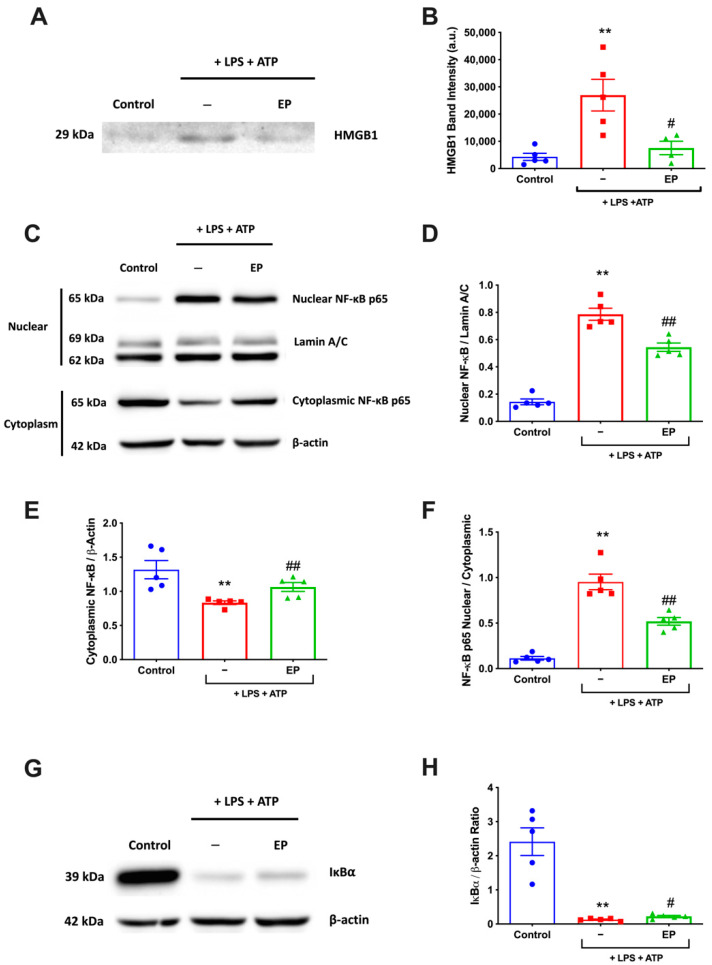
EP suppressed HMGB1 levels and inhibited activation of NF-κB. N9 microglial cells were pretreated with EP (10 mM) for one hour, then treated with LPS (1 μg) for 30 min. (**A**,**B**) EP reduced LPS- and ATP-induced HMGB1 levels. (**C**,**D**) EP pre-treatment reduced phosphorylation of p65 subunit of NF-κB. (**C**,**E**) EP pre-treatment significantly reduced the p50 subunit of NF-κB. (**F**) EP rescued LPS and ATP induced an increase of nuclear/cytoplasmic ratio of NF-κB p65 subunit. (**G**,**H**) EP pre-treatment increased expression of IκBα, which was downregulated after LPS and ATP induction. Data are presented as mean ± S.E.M, n = 5. ** *p* < 0.01 compared to control and # *p* < 0.05 and ## *p* < 0.01 compared to LPS-induced cells.

The effect of EP on HMGB1 and NF-κB signaling was examined with western blotting. Secreted HMGB1 levels in the cell culture media were increased in LPS and ATP treatment group while it was decreased with EP pre-treatment (Figure 5A,B). Furthermore, nuclear NF-κB levels were increased in LPS and ATP group compared to the control group and decreased in EP-pretreated group compared to LPS and ATP (Figure 5C,D). The LPS and ATP treatment increased the HMGB1 and NF-κB levels statistically significantly compared to the control group (*p* = 0.008). Pre-incubation with 10 mM EP significantly decreased LPS and ATP including the HMGB1 and NF-κB levels (*p* = 0.008). EP pre-treatment significantly rescued LPS- and ATP-induced IκBα decrease. IκBα levels were increased in EP pre-treatment group compared to LPS and ATP group (*p* = 0.0286) (Figure 5G,H).

### 3.6. miR-223 Inhibition Attenuates Protective Roles of EP

**Figure 6:**
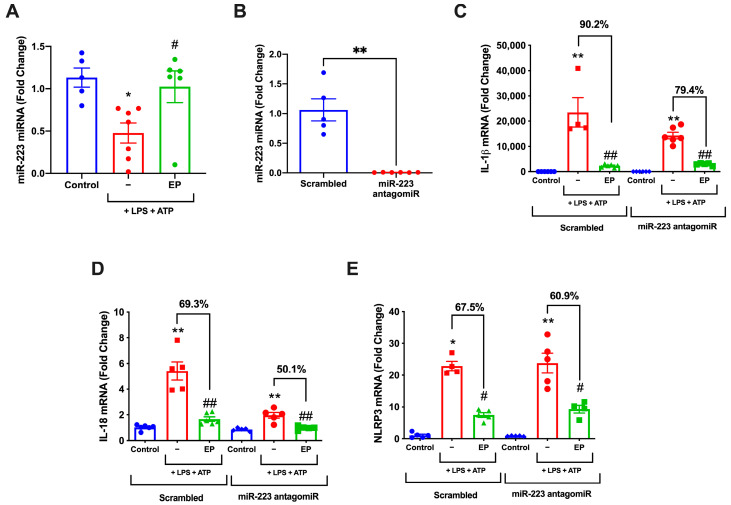
miR-223 inhibition impairs the protective role of EP in inflammasome activation. N9 cells were treated with antagomiR-223 before EP (10 mM) for one hour, then treated with LPS (1 μg/mL) for four hours, and ATP (5 mM) for 1 h. (**A**) miR-223 expression without any modification decreased in LPS- and ATP-treated group and increased in the EP pre-treatment group. (**B**) miR-223 levels were suppressed following miR-223 antagomiR transfection. (**C**) After mir-223 inhibition, the difference in NLRP3 between the LPS and ATP and EP pre-treatment groups decreased. (**D**) After mir-223 inhibition, the difference in IL-1β between the LPS and ATP group and EP pre-treatment group was decreased. (**E**) After mir-223 inhibition, the difference in IL-18 between the LPS and ATP group and the EP pre-treatment group was decreased. Data are presented as mean ± S.E.M, n = 5. * *p* < 0.05 and ** *p* < 0.01 compared to control and # *p* < 0.05 and ## *p* < 0.01 compared to LPS-induced cells.

The effect of EP on miRNA expression was analyzed by qPCR. The miR-223 expression was decreased significantly in LPS and ATP group compared to control group (*p* = 0.0286) and increased in EP pre-treatment group significantly (*p* = 0.0286) (Figure 6A). For the functional assays, transfection with miR-223 antagomiR resulted in 0.006-fold decrease in miR-223 levels (*p* = 0.0043) (Figure 6B). The functional study with inhibition of miR-223 showed the reversal of EP-suppressed IL-1β (Figure 6C), IL-18 (Figure 6D), and NLRP3 expression (Figure 6E) compared to negative controls transfected groups.

## 4. Discussion

Inflammasome activation, a response of the innate immune system, is a mechanism that allows the body to respond effectively to exogenous and endogenous threats following the production of inflammatory proteins [25]. Our study utilized N9 cells, and NLRP3 activation was created by stimulation with LPS and ATP, the well-known and most studied inflammasome model [26]. Upon stimulation of cells with LPS and ATP, NF-κB transcription factor triggers the expression of NLRP3 and pro-IL-1β in the resting state, which NLR serves as the first step for NLRP3 inflammasome activation [26,27]. During the microglial inflammasome activation step, oxidative molecules are also accumulated in cells [28]. After NLRP3 inflammasome activation, pyroptotic cell death occurs due to caspase-1 activation, resulting in impaired plasma membrane integrity [29,30,31] and inflammatory responses simultaneously [32]. Our study revealed that LPS and ATP induction in microglia resulted in upregulation of IL-1β, IL-18, NLRP3, ROS levels, and the pyroptotic cell death resulting from the caspase-1 activity.

EP is a derivative of pyruvic acid with potent antioxidant, anti-inflammatory, and cytoprotective effects [16]. In previous reports, EP has been shown to suppress inflammatory cytokine expression in microglial cells. A study with BV2 microglia has reported that EP treatment suppressed the expression of pro-inflammatory cytokines TNF-α and IL-1β [20]. EP was also found to inhibit cleavage of IL-1β and NLRP3 inflammasome activation in attenuation of microglial activation in a mice model of sepsis-associated encephalopathy [33]. EP also reduced an ROS-related increase in NLRP3 inflammasome activation and IL-1β release in other cell types [34]. EP is a well-known inhibitor of NF-κB, shown in RAW264.7 cells [35]. Nevertheless, the effects of EP on NLRP3 inflammasome activation in microglial cells are unknown. Therefore, in our study, EP’s effect on inflammasome activation induced by LPS and ATP was investigated. Here we report that EP significantly reversed pyroptotic cell death and reduced the amount of IL-1β and IL-18 inflammatory cytokines, which are the markers of NLRP3 inflammasome in N9 microglial cells.

HMGB1 is released from dying cells and acts as a chemokine in inflammation propagation. It mediates microglial activation via NF-κB regulated manner. After binding to TLR4, HMGB1 activates inflammation through NF-κB signaling [36]. Under physiological conditions, HMGB1 is found in the nucleus of the cell. However, HMGB1 translocation occurs from the nucleus to the cytoplasm by secretion of an infectious molecule, such as LPS and ATP, from damaged cells [37]. Excessive accumulation of extracellular HMGB1 in tissue or circulation contributes significantly to the pathogenesis of many inflammatory or autoimmune diseases such as sepsis and colitis [38]. In previous studies, it has been shown that LPS- and ATP-induced HMGB1 release is inhibited in macrophage cultures and endotoxemia models due to genetic deletion of NLRP3 or ASC, which are the main components of the NLRP3 inflammatory model [39]. EP reduced caspase-1 activation, HMGB1 release, and NLRP3 inflammasome activation independently from potassium efflux and preserved mitochondrial integrity in mouse macrophages [37].

MiRNAs are a member of short non-coding RNA families with gene regulatory functions that participate in diverse cellular processes, including inflammation [40]. miR-223 is an essential homeostatic factor abundant in peripheral blood cells [41]. In macrophages, polarization states differ in terms of miR-223 expression; M2 polarization shows increased expression of miR-223, while in M1 polarization, miR-223 is downregulated [42]. Furthermore, miR-223 was previously shown to negatively regulate NLRP3 inflammasome activation in vitro and in vivo [11,43,44]. It was also recently reported that miR-223 directly targets NLRP3 inflammasome to reduce LPS-induced inflammatory responses [45]. In our study, treatment of LPS and ATP resulted in downregulation of miR-223 expression, and EP pre-treatment caused miR-223 upregulation, contributing to the inflammasome suppression. Acting along with EP, miR-223 attenuated inflammatory response in LPS- and ATP-treated groups. Induction or inhibition of miRNA expression upon inflammatory stimuli results in altered biological responses like a pro- or anti-inflammatory response [40,46]. miR-223 limits the inflammatory response to prevent collateral damage [47]. 

The gene encoding miR-223 is located on the X chromosome and is transcribed independently of any known gene. The expression of miR-223 is regulated by three transcription factors, namely C/EBPα, PU.1, and NFI-A [48], and there is not any direct evidence that EP affects these transcription factors. On the other hand, EP may alter miR-223 expression through indirect mechanisms, such as long-non-coding RNAs (lncRNAs) and Nrf2 signaling pathway [49,50,51,52]. Some lncRNAs, such as GAS5, DLX6-AS1, and MEG3, have been reported to modulate miR-223 expression [49,50,51]. The Nrf2 signaling pathway is the main antioxidant signaling pathway under the stress condition, and overexpression of Nrf2 upregulates miR-223 expression [52]. It is also well known that EP activates the Nrf2 signaling pathway [53]. Thus, EP may increase miR-223 expression by activating the Nrf2 pathway.

In the present study, we used N9 microglial cell line for in vitro studies. The immortalized N9 microglial cell line was derived from a mouse brain sharing many features with primary microglia [21]. N9 cells express cell surface markers FcR, Mac-1, and F4/80. Furthermore, it has been demonstrated that immune response against inflammation gave similar results as primary microglia. It has the capacity to give an immune response against inflammatory stimuli (Amyloid-β, TNF, IL-1β) and is able to perform phagocytosis [22]. Thus, the N9 microglia cell proves itself and has been extensively used in neuroimmunology research.

## 5. Conclusions

Our results suggest that miR-223 is involved in the anti-inflammatory effects of EP in N9 microglia. Our study is the first study that shows miR-223 involvement in NLRP3 inflammasome activation via HMGB1/NF-κB activation. Although further investigation is needed to uncover miR-223 action mechanisms, it is a promising target in modifying NLRP3 inflammasome activation, as well as HMGB1 and NF-κB.

## Figures and Tables

**Table 1:** Antibody list.

Antibody	Provider	Catalog Number	Dilution
Anti-IL-1β	Abcam	ab9722	1:1000
Anti-Caspase-1	Abcam	ab1872	1:1000
Anti-NLRP3	Adipogen	AG-20B-0014	1:1000
Anti-HMGB1	Cell Signaling	3935S	1:1000
Anti-NF-κB p65	Santa Cruz	sc-372	1:1000
Anti- IκBα	Cell Signaling	9242S	1:1000
Anti-β-actin	Abcam	ab8227	1:1000
Anti-Lamin A/C	Santa Cruz	sc-20681	1:1000
Anti-Rabbit HRP Secondary	Cell Signaling	7074	1:2000
Anti-Mouse HRP Secondary	Cell Signaling	7076	1:2000

**Table 2:** PCR primer list.

mRNA		Sequence (5′–3′)	Product Length	Accession
IL-1β	Forward	TTCTTTTCCTTCATCTTTGAAGAAG	365 bp	NM_008361.4
	Reverse	TCCATCTTCTTCTTTGGGTATTGTT		
IL-18	Forward	CTTTGGAAGCCTGCTATAATCC	363 bp	NM_008360.2
	Reverse	GGTCAAGAGGAAGTGATTTGGA		
NLRP3	Forward	TGCCTGTTCTTCCAGACTGGTGA	143 bp	NM_145827.4
	Reverse	CACAGCACCCTCATGCCCGG		
GAPDH	Forward	ACCACAGTCCATGCCATCAC	452 bp	NM_001289726.1
	Reverse	TCCACCACCCTGTTGCTGTA		

## Data Availability

The data presented in this study are available within the article and its supplementary material. Other data that support the findings of this study are available upon request from the corresponding author.
